# Cardiovascular state changes in simulated work environments

**DOI:** 10.3389/fnins.2014.00399

**Published:** 2014-12-05

**Authors:** Arjan Stuiver, Ben Mulder

**Affiliations:** ^1^Neuropsychology, Behavioural and Social Sciences, University of GroningenGroningen, Netherlands; ^2^Experimental Psychology, Behavioural and Social Sciences, University of GroningenGroningen, Netherlands

**Keywords:** cardiovascular reactivity, mental workload, state assessment, simulated work, baroreflex

## Abstract

The usefulness of cardiovascular measures as indicators of changes in cognitive workload has been addressed in several studies. In this paper the question is explored whether cardiovascular patterns in heart rate, blood pressure, baroreflex sensitivity and HRV that are found are consistent within and between two simulated working environments. Two studies, were performed, both with 21 participants: one in an ambulance dispatch simulation and one in a driving simulator. In the ambulance dispatcher task an initial strong increase in blood pressure is followed by a moderate on-going increase in blood pressure during the next hour of task performance. This pattern is accompanied by a strong increase in baroreflex sensitivity while heart rate decreases. In the driving simulator study, blood pressure initially increases but decreases almost to baseline level in the next hour. This pattern is accompanied by a decrease in baroreflex sensitivity, while heart rate decreases. Results of both studies are interpreted in terms of autonomic control (related to both sympathetic and para-sympathetic effects), using a simplified simulation of a baroreflex regulation model. Interpretation of the results leads to the conclusion that the cardiovascular response patterns in both tasks are a combination of an initial defensive reaction, in combination with compensatory blood pressure control. The level of compensatory blood pressure control, however, is quite different for the two tasks. This helps to understand the differences in response patterns between the two studies in this paper and may be helpful as well for understanding differences in cardiovascular response patterns in general. A substantial part of the effects observed during task performance are regulatory effects and are not always directly related to workload manipulations. Making this distinction may also contribute to the understanding of differences in cardiovascular response patterns during cognitive workload.

## Introduction

Cardiovascular measures are extensively studied in both laboratory and applied environments to gain insight in either responsiveness or in operator state changes during continuing mental work. In recent years, research on operator state assessment has frequently focused on developing applications for adaptive automation. Cardiovascular measures are mentioned by different authors as good candidates for the assessment of operator state because they can be measured relatively easily and continuously (Hockey et al., 2003; Mulder et al., 2003, 2004).

In several studies it has been shown how psychophysiological measures can be used in adaptive automation (Pope et al., 1995; Prinzel et al., 2000; Fairclough and Venables, 2006; Ting et al., 2010). Knowledge about a person's current state, in combination with information about task load and task performance, can be applied to adapt the working environment to fit the user's demands or needs (Haas and Hettinger, 2001). In this context, it is important to take into account the demand a task is placing on the user. The basic idea of adaptive automation or adaptive support is to design a system that will give valuable help, being a companion for the operator during periods of expected over- or underload (Hoogeboom and Mulder, 2004), for example by controlling task demands.

Physiological information that can be used in adaptive automation may consist of response patterns to momentary changes in task load or could consist of state changes related to continuous work or both. In this context, a reaction to changes in workload that last only a short period of time, e.g., 30 s to 1 min, is (part of) a response pattern to momentary changes. State changes indicate the long-term effects up to a couple of hours. One of the prerequisites for using either short-term response patterns or state changes is that consistent cardiovascular patterns have to be available at an individual level for the task under focus. Another requirement is that a distinction between periods of low and high workload can be made on the basis of changes found in these patterns. Knowing that this is a multidimensional problem, the focus of the present paper will be on the consistency of cardiovascular state changes in two task environments: an ambulance dispatching task and a driving simulator. Based on the results found suggestions will be made on how to apply such measures and which issues have to be addressed for applications in the world of adaptive automation.

Effects of mental effort on heart rate (HR) and heart rate variability (HRV) have been extensively studied during laboratory tasks (Mulder and Mulder, 1987; Backs and Seljos, 1994), simulated work (Brookings et al., 1996; Veltman and Gaillard, 1998; De Rivecourt et al., 2008; Dijksterhuis et al., 2011) and during real work (Roscoe, 1992; Wilson, 1993; De Waard et al., 1995; Hankins and Wilson, 1998). In general, during effortful working periods in these tasks, a pattern is found of increased HR in combination with decreased HRV, compared to resting baselines or compared to conditions of lower workload.

However, the sensitivity of these effects depends strongly on the task load differences between conditions, the type of task and time on task (Mulder and Mulder, 1987; Althaus et al., 1998; Backs and Boucsein, 2000). Effects of increased task demand correspond with elevated (finger) blood pressure (BP), lowered baroreflex sensitivity (Mulder and Mulder, 1981; Reyes del Paso et al., 2004) and higher respiration rate (Wientjes, 1992). Some authors have characterized this response as a defense reaction or a preparatory fight-or-flight response (Mulder, 1980; Jordan, 1990; Berntson et al., 1991). Generally speaking, the effects in laboratory tasks and simulated work are more transparent than those in real life work environments, due to varying task demands, unknown task characteristics, time on task effects and adaptivity of the short term blood pressure control system (baroreflex, e.g., Mulder, 1992) to these changing task demands. In laboratory conditions HRV clearly showed more consistent effort effects than HR and is therefore proposed as a good indicator of invested mental effort in cognitively demanding tasks (Mulder and Mulder, 1981).

A well-known fatigue and monotony effect on HR, i.e., a gradual decrease during the working day, is reported in many studies (Myrtek et al., 1994; Raggatt and Morrissey, 1997). A similar phenomenon is found during much shorter sessions, e.g., an hour of continuous task load. Mulder et al. (1993) showed that initial HR(V) effects resembling a defense reaction disappeared after 10–20 min in a memory search task that lasted 45 min, while BP and baroreflex sensitivity (BRS) remained the same after the initial effects, i.e., BP remained high and BRS remained low. The authors concluded that these effects were directly related to short-term blood pressure control (baroreflex, Van Roon et al., 2004; Mulder et al., 2009), a control system in the body that tries to maintain stable blood pressure. Baroreceptors monitor changes in blood pressure and influence sympathetic and para-sympathetic activation. A continuing elevation of blood pressure increases sensitivity of this system and increases parasympathetic and decreases sympathetic activation, lowering heart rate and indirectly blood pressure. The question arises how generalizable and how consistent the patterns described above (an initial defense response followed and possibly overshadowed by effects of the short-term blood pressure regulation mechanism) actually are and how to interpret such changes in terms of autonomic control (Berntson et al., 1991; Backs, 1998).

The effects of the typical adaptive baroreflex response described above may obscure the direct effects of task demand manipulations, because these adaptive effects might overshadow or at least diminish the effects of changes in task demand. It has to be kept in mind that both types of effects occur simultaneously and are in principle present at any moment, which makes interpretation of effects of task demand more difficult. This may be a reason to split the research regarding these effects in two directions: the first perspective is to look at specific cardiovascular state changes over longer time periods. The second is to look at smaller time segments connected to specific short lasting changes in task demand. In this paper we will focus on the first approach.

One of the two environments was an ambulance dispatchers' simulation, in which the participants' main task was to send the ambulances to accidents or schedule them for non-emergency rides (Blandford and Wong, 2004; Mulder et al., 2009). Most of the workload was related to the cognitive aspects of the task, such as perceiving the environment and keeping an up to date model of what is happening at every moment. Handling the telephone calls, planning scheduled rides and deciding which ambulances to use also placed strain on the participant. Because it was a computerized task the work required little physical effort. Together with the time pressure in some periods, working memory aspects were the main contributing factors to the workload the participant experienced. In conclusion, the planning of non-urgent rides and keeping coverage of the region may be seen as a continuous loading task that was interrupted every now and then (increasing task difficulty) by incoming emergency calls.

The other environment was a fixed-base high fidelity driving simulator. In this study participants had to drive eight road sections on which they had to pass crossroads with heavy traffic. Driving in a simulator and more specifically passing intersections is predominantly a cognitive task. Monitoring traffic and deciding when to cross imposes more mental than physical load. There was, however, a higher degree of physical activity and less cognitive activity involved in the driving task compared to the dispatcher task. Participants had to steer and use the pedals, which may have evoked some minor physical load effects as well.

The studies reported here can be compared to previous research. Mulder et al. (2009), using the same ambulance dispatcher environment, found a cardiovascular pattern as a function of time of an ongoing increase in blood pressure in combination with decreased HR and baroreflex sensitivity, while HRV in the mid frequency band (0.07–0.14 Hz) increased as well as function of time. No (continuous) blood pressure data from former driving simulator experiments are available. HR and HRV data are available however. The main findings are that HR effects as a function of task load are in general stronger (larger increases) than those of HRV (expected decreases). The same pattern of effects was found in simulated flight (Veltman and Gaillard, 1998; De Rivecourt et al., 2008). Since flying also requires physical activity to a certain degree, this may be linked to the motor aspects of the task.

As described above, based on previous research, an increase in blood pressure over time and an accompanying lowering of heart rate and increase in heart rate variability can be expected in the dispatcher task. Since there is little explicit information about changes in blood pressure during driving, the same time course of changes in blood pressure, heart rate, baroreflex sensitivity and HRV may be expected to occur during driving as found in the ambulance dispatcher task.

To conclude this introduction the following research questions can be formulated:

- Can a consistent pattern of state changes be found for each of the two experimental task environments and are these patterns comparable?
- Can the patterns be explained in terms of cardiovascular regulation, including changed autonomic activation as a function of time?
- What can be concluded with respect to the usability of patterns of cardiovascular state changes for operator state assessment?

## Methods

The main goal of the present paper was to compare the patterns of cardiovascular state changes in two different simulated working environments: an ambulance dispatcher task and a driving task. In each of these environments an experiment has been conducted. In this section, the experimental settings and procedures are described for each of the experiments separately, while the common (data analysis) parts are described together at the end of this section.

### Ambulance dispatcher task

#### Participants

A total number of 22 participants (between 19 and 27 years of age, 12 female, all students) took part in the experiment. The data of 21 were used for analysis. The results of one participant were excluded due to difficulties with blood pressure measurement. All participants were students of the University of Groningen. They received a financial reward for their participation and signed an informed consent at the start of the first training session. The study was approved by the ethical committee of the faculty of Behavioural and Social Sciences at the University of Groningen.

#### Dispatcher task

Participants had to perform three main tasks. Firstly, they had to activate emergency rides as a response to emergency calls. They had to select the optimal or most economic ambulance, which might have been an ambulance driving in the vicinity of where the emergency occurred or an ambulance from a post nearby. To make this choice, the participant needed a good overview of the current location of ambulances and the current situation in the region. The second task was scheduling ambulances that carried out non-urgent transport rides, transporting patients to and from hospitals as scheduled. In more detail: non-urgent rides were either in the “transport-list” at the beginning of each scenario or would come in during task performance as a telephone call (non-urgent). This part of the task required a lot of scheduling and planning. The last part of the task was to make sure that every place in the entire area could be reached by an ambulance within 15 min. To preserve this coverage the participant had to choose optimal ambulances for emergency rides and non-urgent transport. This also required good insight in the location and activities of the ambulances.

In this experiment the dispatch center was simulated on a computer with two screens. On one screen the communication and planning interface was shown. Participants used a regular mouse and keyboard as interface. On the other screen a map of the region was shown on which ambulances (moving) and hospitals, ambulance stations and emergency locations (stationary) were represented. Communication was achieved through screen messages to keep the system uniform and suitable for experiments.

#### Training

Participants completed an extensive training period of 6 h in three sessions. One of the goals of the training was learning the topography of the province of Groningen used in the task. Participants were trained in knowing the location of the most important places and the time it would take ambulances to drive between these places. However, if during the experiment a participant could not remember where a city was located or how long it would take an ambulance to reach a destination, he or she could use an interactive map included in the task interface to find that information.

A second goal of the training was to learn how to perform the task, i.e., how to use the application, find ambulances on the map, find cities and driving times between places, how to dispatch ambulances, plan non-emergency rides, and keep coverage. Participants were trained gradually and their progress was evaluated at several moments. Toward the end of the training period the scenarios became more realistic and were more similar to and at the same complexity-level as the scenarios used in the experiment. Since the simulated task consisted mainly of the planning aspect of the real dispatcher task, planning was the focus point of training and testing. Therefore, after training all participants were tested on their topographical knowledge and their skill in estimating how much time it would take to drive from one place to another. If they did not attain an acceptable level (60% correct), they were asked to spend extra time on training and were re-tested before the experiment. Of course, participants reveiced a less elaborate training compared to real dispatchers, and their training was only sufficient due to the simplification of the task.

#### Experimental procedure

Participants attended two experimental sessions on two different days; each took about 3 h, including a break of 15 min in the middle. The experimental session started with a 5-min baseline (Rest), four scenarios (each lasting 15 min), a break of 15 min, 5-min Rest measurement, and again four scenarios of 15 min each.

In half of the scenarios all the participants had to do was to respond to emergency calls and keep coverage. The scheduled rides were planned and activated “automatically” in such a way that they did not need to be activated during the scenarios and could effectively be ignored. In the other four scenarios they had to plan and activate the scheduled rides and to respond to emergency calls while guarding coverage. The order of presentation of scenarios was balanced to prevent order effects, as they will average out in the results. Participants were randomly assigned to conditions.

### Simulated driving task

#### Participants

From an initial number of 23 participants, 20 finished the experimental sessions (age between 19 and 25 years, 9 female). Participants were required to have held their license for at least a year and had driven at least 5000 km. They received a financial reward for their participation. At the start of the experiment they had to fill in a questionnaire about their age and driving experience and signed an informed consent. The study was approved by the ethical committee of the faculty of Behavioural and Social Sciences of the University of Groningen.

#### Virtual driving environment and task

The study was conducted using a ST Software^©^ driving simulator, consisting of a fixed-base vehicle mock up with functional steering wheel, indicators, and pedals. The simulator was surrounded by three 32″ diagonal plasma screens. Each screen provided a 70° view, leading to a total 210° view. A detailed description of the driving simulator functionality can be found in Van Winsum and Van Wolffelaar (1993). Participants steered with only the right hand to allow taking finger blood pressure measurements on the other hand. For the same reason the simulator car had automatic transmission.

Participants completed a route with a total of four rural and urban areas with either traffic from both sides on crossings or from one side. Participants drove the same track twice, thus driving a total of eight sections that took about 10 min to complete when driving at an average speed of 80 km/h. An urban segment with traffic coming from both sides on the crossing was followed by a rural section also with traffic from both sides, thereafter an urban section with traffic coming only from the right side on the crossings and finally a rural section with traffic also only from the right. Participants were selected randomly to start at one of the four segments. The starting locations were balanced to prevent order effects.

In each section there were six crossings, without road-priority, while normal European traffic rules had to be followed. Other road characteristics such as lane width, curvature, number of lanes (one for each direction) were not different for the sections.

#### Experimental procedure

Participants completed one experimental session, which took about 2.5 h; in contrast to the dispatcher task there was only a 5-min rest measurement and no further break in the middle. The experimental session started with a short training ride of about 10 min to get acquainted with driving in the simulator. After that, there was a 5-min baseline (Rest), four road sections (lasting for about 10 min each), 5-min Rest, and another four road sections. After each road section participants were asked by a computerized voice to stop at a lay-by to report their invested mental effort during the preceding road section on the Rating Scale Mental effort (RSME, Zijlstra, 1993). After four sections, participants had completed one round, after which they drove the same round again, meaning that they drove a total of eight sections. These eight sections were the driving task equivalent of the eight scenarios created for the dispatcher task.

#### Cardiovascular measures

Cardiovascular measurement and analysis procedures were similar for the two experiments and will be described together in this section.

The electrocardiogram (ECG) was recorded with three Ag-AgCl electrodes. The common electrode was placed at the sternum and the other two electrodes at the right and left side between the two lowest ribs. Blood pressure was measured with a FIN.A.PRES device (Finometer®). Both ECG and blood pressure were sampled at 250 Hz. R-peaks were detected online from the ECG by using a hardware ECG-trigger as part of a TMSi Porti measuring system (Twente Medical Systems International). Interbeat intervals (IBI's) were derived from R-peak time points and automatically corrected using the CARSPAN program (Mulder, 1992), followed by visual inspection and manual correction where necessary.

Spectral analysis of all cardiovascular data was also done with CARSPAN, as well as calculation of mean values of heart rate and systolic blood pressure. Spectral HRV values, on the basis of heart rate changes, were only derived for the mid frequency band (0.07–0.14 Hz) based on previous findings in the dispatcher task environment (Mulder et al., 2009). HRV estimates were calculated as values normalized to the mean, i.e., modulation index (Mulder, 1992; Veldman et al., 1998). The variables were logarithmically transformed to obtain normally distributed variables (Van Roon et al., 2004). An index of baroreflex sensitivity (BRS) was created by calculating the transfer gain (modulus) from systolic blood pressure changes to interbeat interval changes in the mid frequency range (Robbe et al., 1987).

#### Applying a baroreflex simulation model

For estimation of (para-)sympathetic effects of workload in the simulated working environments we did a simplified simulation study using a baroreflex model. This model was initially developed by Wesseling and colleagues (Wesseling and Settels, 1985) and further extended, tested and configured for mental workload studies by van Roon (1998). In this model the basic mechanisms of short-term blood pressure control are implemented, such as baroreflex function, and effector systems like heart function (rate and contraction force), peripheral resistance changes and venous filling of the heart. The main assumptions in this model-approach are that after tuning model-parameters to a baseline measurement, all subsequent adaptations during task performance are related to sympathetic and vagal control. Van Roon et al. (2004) extensively described this model, including some working examples. A complete application study was performed by Althaus et al. (2004).

It would be beyond the scope of this paper to perform and describe complete simulations for the current data sets. Therefore, a simplified procedure is applied, making use of the main characteristics of the model. One of these characteristics is that mean blood pressure is nearly independent of vagal activation and therewith almost completely determined by sympathetic control (Van Roon et al., 2004). With this knowledge, sympathetic gain changes in respect to baseline can be determined. Having this information, subsequently, changes in vagal gain can be resolved from HR data. Finally, the obtained vagal and sympathetic gain estimates can be checked against the measured BRS and HRV values.

#### Variables, experimental design and statistical analysis

In the ambulance dispatcher task, analysis periods of 5 min were selected; for each of the scenarios three such periods were averaged to get one value per scenario. Taking averages of spectral values over 5 min periods instead of one spectral value for the total 15 min scenario, helps overcome the problems that may arise from non-stationarities in the signal (Weber et al., 1992). A total of 10 values were obtained per variable, per session (two resting periods, eight scenarios). Data of the two sessions were averaged. For the driving task, analysis periods of 10 min were used for the driving segments and periods of 5 min for the resting phase. This resulted for the driving task also in 10 values: two resting periods, eight road segments.

Analyses on the following variables will be reported: mean heart rate (in beats/minute), mean systolic finger blood pressure (in mmHg), HRV values from the mid-frequency band (in natural log-transformed squared modulation index values), BRS in the mid-frequency band (in ms/mmHg) and a rating of subjective mental effort on the RSME (Zijlstra, 1993).

The data were analyzed using the General Linear Model Repeated Measures test in SPSS. The same design was applied for both experiments. Repeated Measures MANOVAs were run on all the four variables (heart rate, systolic blood pressure, baroreflex sensitivity and heart rate variability) simultaneously. Values for the first rest period vs. the second rest period were compared. Rest vs. task effects (two levels; first rest vs. task and second rest vs. task) were tested using the data from the first task segment following the rest period. It has to be noted that in the dispatcher task the first 5-min segment of the first scenario after a rest measurement was used, while for the driving experiment the first road segment (10 min) after a rest measurement was applied. Level differences as well as trend lines were tested for the first and the second task part (first four scenarios vs. the last four, first four road segments vs. the last four). This resulted in 6 different tests for each variable, for the resulting familywise error rate a corrected alpha, originally set to 5%, was calculated with the Holm-Bonferroni method (Holm, 1979). In the result section it is indicated if the *p*-value was larger than the corrected alpha and the result therefore not statistically significant.

## Results

### Ambulance dispatcher task

**Figure 1:**
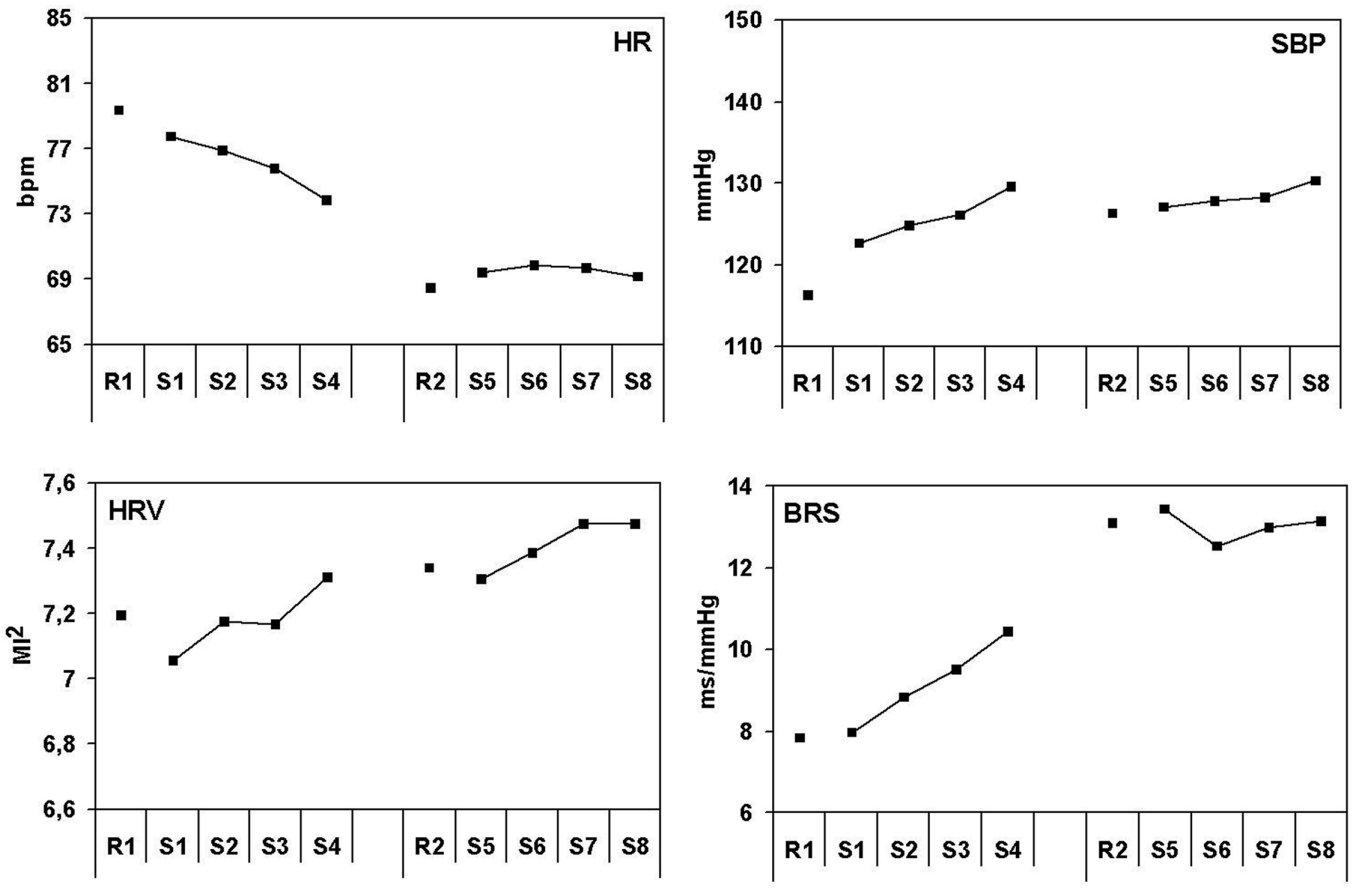
**Effects of lasting task demands on cardiovascular measures in the ambulance dispatchers' task**. Heart rate in beats per minute, systolic blood pressure in millimeters Mercury, heart rate variability in squared modulation index and baroreflex sensitivity in milliseconds per millimeter Mercury. R1, rest period 1; R2, rest period 2 and S1–S8 correspond with scenario 1–8.

Figure 1 shows the response patterns for HR, SBP, HRV, and BRS for the dispatcher task. Large changes only occur in the first part of the session, while values remain at the same (extreme) level after the first part. HR strongly decreases over the session, from about 80 beats per minute during the first rest, to about 70 beats/min during the last scenarios. Statistical analysis shows that there are no rest-task differences. There is a decrease in HR during the first half of the session [*F*_(1, 20)_ = 39.6, *p* < 0.001], while the level does not change in the second half of the session. Overall, the HR level of the second part of the session is lower than the first part [*F*_(1, 20)_ = 160.2, *p* < 0.05]. HR during the first rest period is lower than during the second one [*F*_(1, 20)_ = 76.7, *p* < 0.001].

Systolic blood pressure increases strongly during the first scenario, compared to the preceding rest (about 7 mmHg). This increase continues during the first scenarios and SBP reaches very high values in the last scenario before the pause (about 15 mmHg higher compared to the first rest). After the pause, SBP stays at a high level and increases only slightly further. Results from statistical tests confirm an on-going blood pressure increase from rest to the first task [*F*_(1, 20)_ = 15.6, *p* < 0.05], an ongoing increase in the first part [*F*_(1, 20)_ = 13.1, *p* < 0.05], and a SBP difference between the first and the second rest measurement [*F*_(1, 20)_ = 16.4, *p* < 0.001]. Finally there is a small but significant ongoing increase in the second part of the task [*F*_(1, 20)_ = 8.01, *p* < 0.05].

Baroreflex sensitivity shows more or less the opposite effect from heart rate. BRS increases strongly during the first part of the session (about 3 ms/mmHg) and stays at an even higher level after the pause, during both the rest and the subsequent scenarios (a very large (significant) difference of about 7 ms/mmHg with the rest at the start of the session). A large difference in BRS between the first and the second rest measurement [*F*_(1, 20)_ = 76.7, *p* < 0.001] was found, in combination with a clear increase [*F*_(1, 20)_ = 39.6, *p* < 0.001] of BRS during the first part of the session and no further increase in the second part. There was no initial rest-task difference, while the BRS level was distinctly higher during task performance after the pause than before [*F*_(1, 20)_ = 61.6, *p* < 0.05].

HRV shows a gradual increase during the session: a linear increase in HRV can be seen during the first [*F*_(1, 20)_ = 29.4, *p* < 0.001] and the second [*F*_(1, 20)_ = 9.1, *p* < 0.05] part of the session. The HRV level was lower in the first than in the second part of the session [*F*_(1, 20)_ = 13.7, *p* < 0.05].

Participants reported a higher rating of subjective effort in the first session compared to the second [*F*_(1, 20)_ = 18.46, *p* < 0.001] with an average for both sessions of 45.8. A difference of 16.6 between easier and more difficult scenarios was also found, with 37.5 for the easier scenarios and 54.1 for the other [*F*_(1,20)_ = 49.50, *p* < 0.001]. The results are summarized in Table 1.

**Table 1:** **Results of the ambulance dispatcher's study**.

	**Heart rate**			**Systolic blood pressure**		
	***F*_(1, 20)_**	***p*-Value**	**η^2^_p_**	***F*_(1, 20)_**	***p*-Value**	**η^2^_p_**
Rest1 vs. Rest2	92.6	<0.001	0.82	18.2	<0.001	0.49
Rest vs. Task (1st)	2.83	0.11	0.12	17.3	0.001	0.48
Rest vs. Task (2nd)	3.95	0.061	0.17	0.20	0.65	0.011
1st Half vs. 2nd Half	197.3	<0.001	0.91	1.70	0.21	0.083
Lin. Trend 1st Half	31.4	<0.001	0.61	14.6	0.001	0.44
Lin. Trend 1st Half	0.35	0.56	0.017	8.89	0.008	0.32
	**Baroreflex sensitivity**			**Heart rate variability**		
Rest1 vs. Rest2	84.0	<0.001	0.82	5.06	0.036	0.20
Rest vs. Task (1st)	0.13	0.73	0.007	4.16	0.055	0.17
Rest vs. Task (2nd)	0.80	0.38	0.040	2.36	0.14	0.11
1st Half vs. 2nd Half	68.4	<0.001	0.78	17.9	<0.001	0.47
Lin. Trend 1st Half	42.3	<0.001	0.69	31.8	<0.001	0.61
Lin. Trend 1st Half	0.19	0.67	0.010	8.7	0.008	0.30

### Simulated driving task

**Figure 2:**
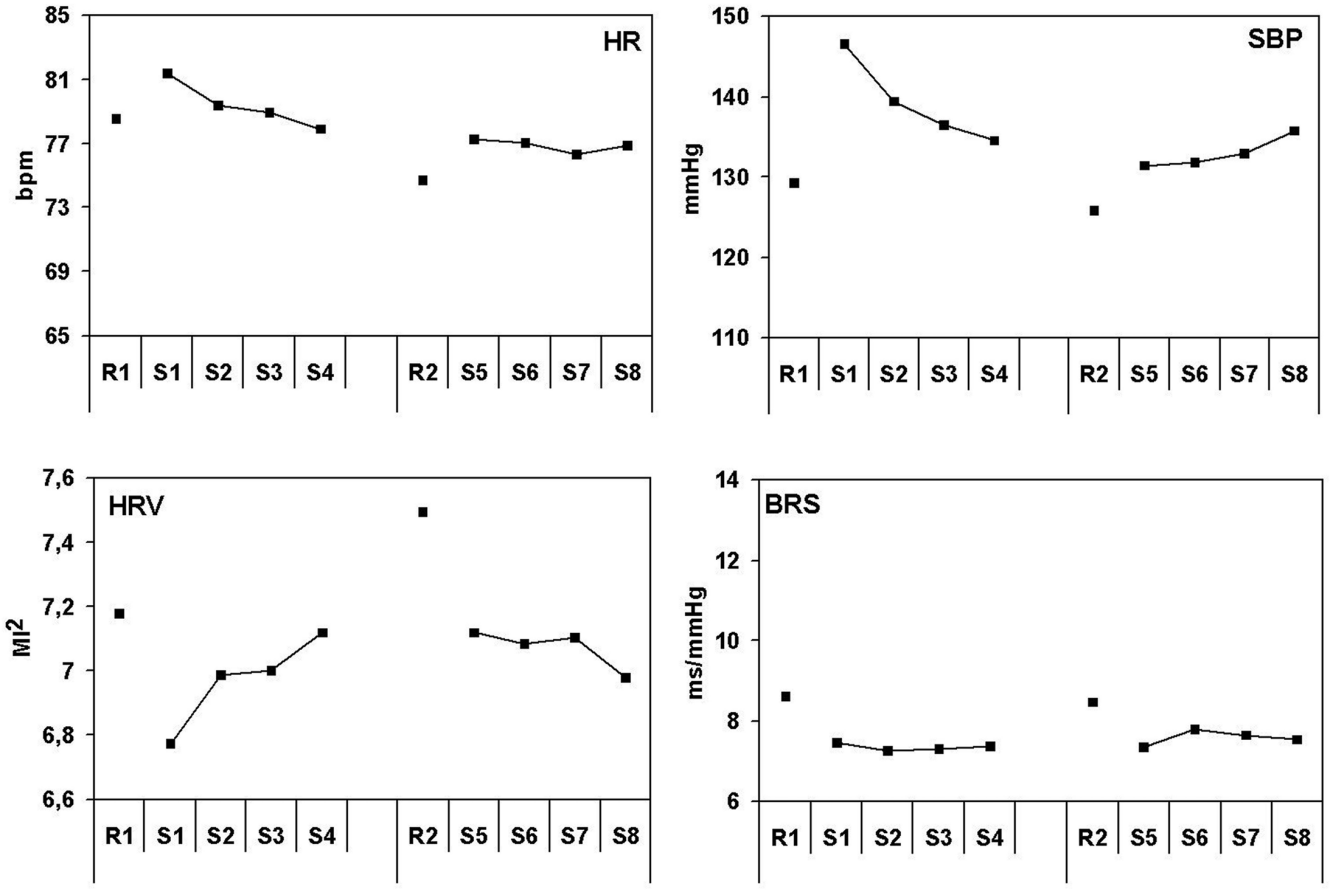
**Effects of lasting task demands on cardiovascular measures in the driving simulator**. Heart rate in beats per minute, systolic blood pressure in millimeters Mercury, heart rate variability in squared modulation index and baroreflex sensitivity in milliseconds per millimeter Mercury. R1, rest period 1; R2, rest period 2 and S1–S8 correspond with scenario 1–8.

Figure 2 shows the response patterns for HR, SBP, HRV, and BRS for the simulated driving task. The overall time course of the cardiovascular variables in the driving task is quite different from that in the dispatcher task. This holds in particular for SBP and BRS.

The heart rate pattern can be characterized by an initial increase during driving compared to the preceding rest in both the first and the second part of the task. The initial increase is followed by a gradual decrease, which is strongest in the first part of the session. Heart rate is lower during the second part of the session, both during rest and driving. Statistical analysis confirms the initial HR increase from rest to task [first part: *F*_(1, 19)_ = 8.19, *p* < 0.05] and the gradual decrease during driving [*F*_(1, 19)_ = 14.7, *p* < 0.05] in the first part. HR is indeed lower during the second rest [*F*_(1, 19)_ = 13.9, *p* < 0.05] and the second part of the task [*F*_(1, 19)_ = 21.1, *p* < 0.001] compared to the corresponding periods in the first part of the session.

The blood pressure pattern resembles the heart rate pattern to a large extent. There is a very strong initial increase of SBP (about 17 mmHg) in the first part of the driving task compared to the preceding rest. SBP level shows a gradual and strong decrease during the first part of the driving task (more than 10 mmHg). In the second part of the task the pattern is quite different: a smaller initial increase after rest is found, as well as a small gradual increase during driving. Statistical analysis confirms the initial rest—task difference [first part: *F*_(1, 19)_ = 63.4, *p* < 0.001; second part: *F*_(1, 19)_ = 19.6, *p* < 0.001] and the SBP decrease in the first part [*F*_(1, 19)_ = 34.6, *p* < 0.001].

BRS results only shows trends for rest task differences that are not statistically significant (after Holm-Bonferroni corrections), for both the first part [*F*_(1, 19)_ = 5.16, *p* < 0.035] and the second part [*F*_(1, 19)_ = 4.16, *p* = 0.056]. There were no other effects on BRS.

HRV shows a gradual increase during the first part of the session [*F*_(1, 19)_ = 19.6, *p* < 0.001]. HRV does not seem significantly higher in the second part, when compared with the first part of the session nor does it seem to increase within the session.

Subjective mental effort measured by RSME shows no effects over time. The average level of rated effort was 40.8, with a difference of 7.3 between easier and more difficult sections [*F*_(1, 19)_ = 4.8, *p* < 0.05]. The results of the driving simulator study are summarized in Table 2.

**Table 2:** **Results of the driving simulator study**.

	**Heart rate**			**Systolic blood pressure**		
	***F*_(1, 19)_**	***p*-Value**	**η^2^_p_**	***F*_(1, 19)_**	***p*-Value**	**η^2^_p_**
Rest1 vs. Rest2	14.0	0.001	0.42	1.08	0.31	0.054
Rest vs. Task (1st)	8.19	0.010	0.30	63.4	<0.001	0.77
Rest vs. Task (2nd)	5.22	0.034	0.21	19.6	<0.001	0.51
1st Half vs. 2nd Half	21.1	<0.001	0.52	4.56	0.046	0.19
Lin. Trend 1st Half	14.7	0.001	0.43	34.6	<0.001	0.65
Lin. Trend 2st Half	0.44	0.51	0.023	6.03	0.024	0.24
	**Baroreflex sensitivity**			**Heart rate variability**		
Rest1 vs. Rest2	0.084	0.78	0.004	7.88	0.011	0.29
Rest vs. Task (1st)	5.16	0.035	0.21	5.68	0.028	0.23
Rest vs. Task (2nd)	4.16	0.056	0.18	6.89	0.017	0.27
1st Half vs. 2nd Half	0.56	0.47	0.028	1.91	0.18	0.091
Lin. Trend 1st Half	0.024	0.88	0.001	19.6	<0.001	0.51
Lin. Trend 2st Half	0.10	0.75	0.005	3.13	0.093	0.14

#### Results of the baroreflex model simulations

**Figure 3:**
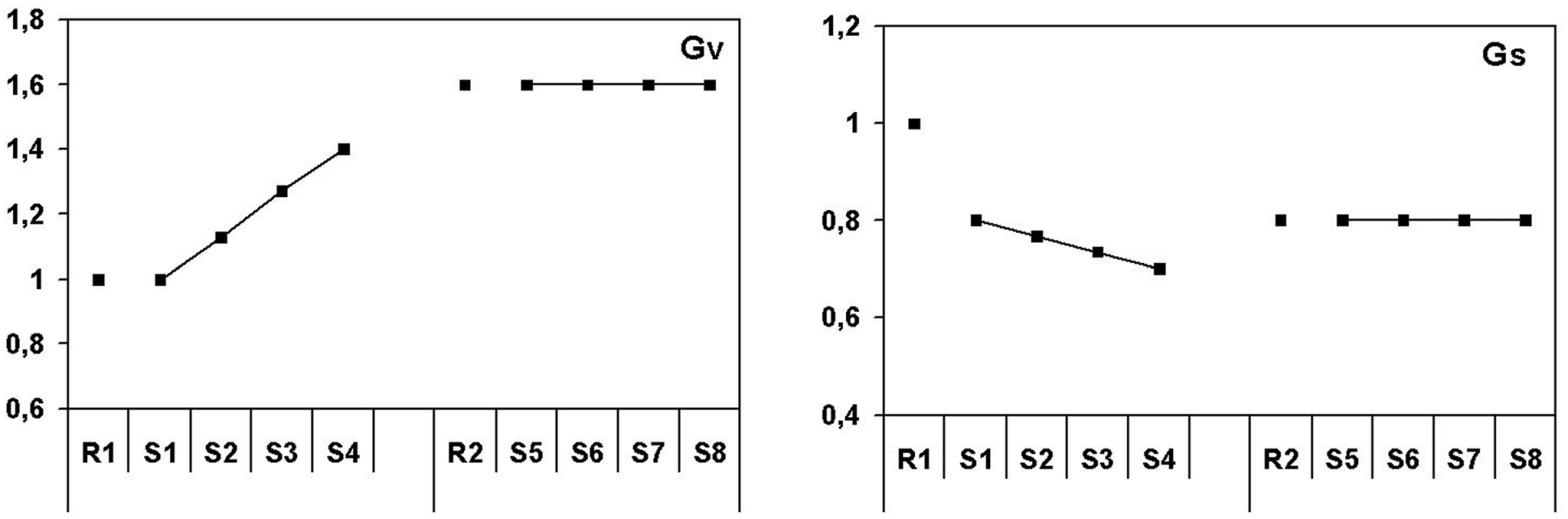
**Patterns of autonomic activation for the ambulance dispatch center simulation study**. Sympathetic and vagal gain in normalized units.

The results of the baroreflex model simulation are depicted in Figure 3. For the ambulance dispatcher task sympathetic gain decreases 20% in the first 15 min compared to the baseline. This can be derived from the 20% increase in blood pressure from the first rest to the first task (Figure 1). It is important to note that although it sounds illogical, both in the model and in real life a decrease of sympathetic gain corresponds with increased sympathetic activity. This inverse relationship does not occur for the vagal control loop. After the initial decrease in sympathetic gain, a further decrease toward 30% occurs during the remaining first hour of task performance. In the second half of the task sympathetic gain stays 20% decreased compared to baseline. From the relationship between vagal gain, sympathetic gain and heart rate, given by the model, it can be derived that vagal gain does not change in the first 15 min. The decrease in heart rate from rest to task is therefore in this case mainly due to sympathetic gain changes. The same relationship given by the model suggests that the lower heart rate during task performance is due to an increase in vagal gain, partly compensated by a decrease in sympathetic gain. More specifically, vagal gain increases strongly and gradually with 40% in the remaining part of the first hour. In the second hour it remains constant at an even higher level of about 60% compared to baseline.

For the simulated driving task, the initial decrease of sympathetic gain is strong, between 35 and 40% as derived from the increase in blood pressure. The large decrease diminishes gradually in the remaining part of the first hour toward 20%. In the second rest measurement sympathetic gain returns to baseline (even 5% higher), while during the second half of the driving task this level is decreased with about 20%. After the first 15 min, vagal gain shows quite a different pattern compared to the ambulance planning task. During the first 15 min vagal gain does not change compared to baseline (changes in heart rate are again mainly due to sympathetic changes). Toward the end of the first driving hour it gradually decreases with about 15%, when the effects of vagal gain are almost totally compensated by sympathetic changes which are indicated by heart rate returning to its original level. During the subsequent resting period, vagal gain returns to baseline level (even 5% higher) when blood pressure is at or below baseline and heart rate as well. It decreases when driving starts again to stay at a constant level of 20% gain reduction indicated by a rise in both blood pressure and heart rate.

## Discussion and conclusion

The main research questions were: can we find a consistent pattern of state changes, will these patterns be similar in different test environments, can these patterns be explained in terms of cardiovascular regulation and what can be concluded with respect to the usability of such patterns for operator state assessment?

Two characteristic, but different cardiovascular patterns were found as a function of time-on-task for the two different tasks. The pattern of the ambulance dispatcher task can be characterized by a small but distinct initial increase in blood pressure, followed by an on-going increase during the first hour of task performance. The break did not reduce this level and blood pressure remained high during the second hour. This pattern was accompanied by strongly reduced heart rate and extremely increased baroreflex sensitivity. The whole pattern may be summarized as a relatively small initial task effect (defense/fight or flight response) followed by strong regulatory effects of baroreflex short-term blood pressure control.

**Figure 4:**
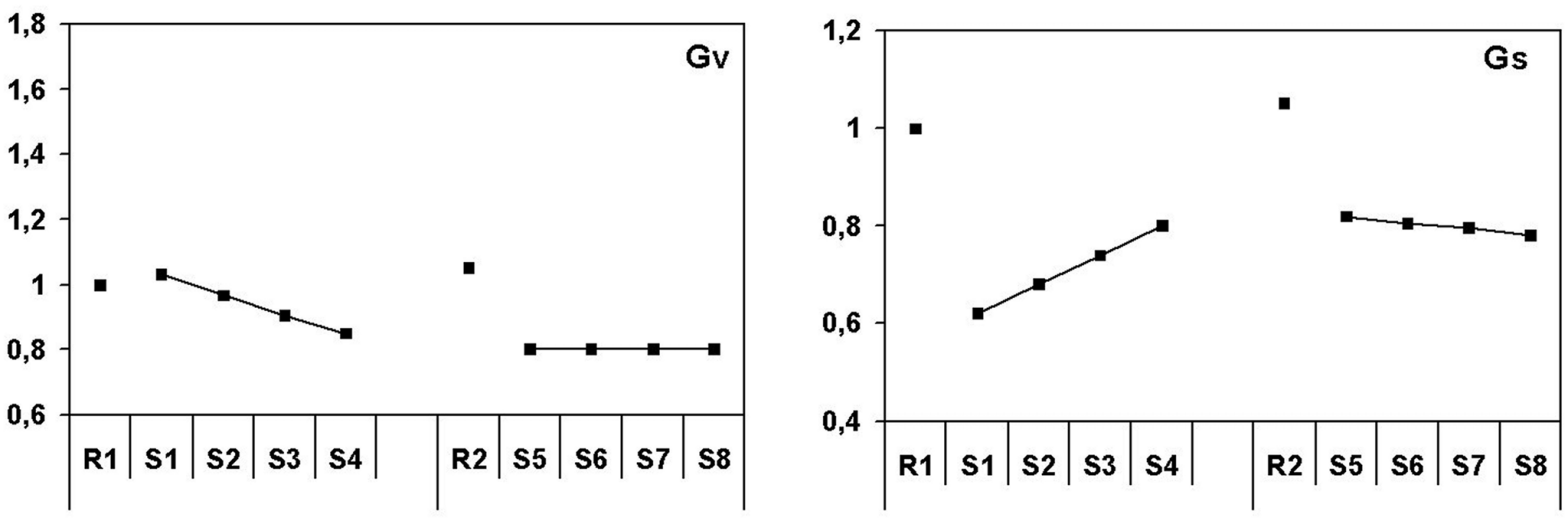
**Patterns of autonomic activation for the driving simulator study**. Sympathetic and vagal gain in normalized units.

The patterns found in the driving task (Figure 4) have other characteristics than those found in the dispatcher task. The initial effects are stronger, while no ongoing increase in blood pressure is seen, which is reflected in a quite different baroreflex pattern. A strong initial increase of systolic blood pressure in the first 10 min of task performance is followed by an on-going decrease. The initial increase is reduced with more than 50% in the subsequent hour. The heart rate pattern resembles the blood pressure pattern, with the exception that the decrease in heart rate was stronger. Remarkable is the baroreflex pattern that shows no significant initial rest–task differences and that remains at the same level during each of the two driving hours. BRS seems to have much lower values during driving than in the ambulance dispatcher's task, though this has not been tested. The magnitude of change in the ambulance dispatcher's task is quite large with 5.5 ms/mmHg, in the driving task it is not significant with 1.2 ms/mmHg.

The question arises what the reason is for these different time-on-task cardiovascular patterns for these two task environments and whether similar patterns have been found in other, comparable studies. First of all, reported mental effort was higher in the first session of the dispatcher task and decreased over time. This decrease was not found in the driving task. The difference between easier and more difficult scenarios was larger in the dispatcher task and on average higher, suggesting that workload was higher in the dispatcher task and even more so in the more difficult scenarios. This might partly explain the differences in baroreflex patterns between the two tasks.

The ambulance dispatcher task has been applied in a series of experiments in our laboratory in recent years. Mulder et al. (2009) reported two of these studies. The first showed the same pattern of results in an ambulance dispatcher task with alternating easy and difficult task periods, lasting for about 2 h. Heart rate and baroreflex changes were of the same magnitude as in the present experiment, while blood pressure changes were even somewhat larger. Results of the second study reported in Mulder et al. (2009) also showed the same pattern in a 1 h lasting task period, but the magnitudes of the responses were slightly lower for all variables, including systolic blood pressure, heart rate and baroreflex sensitivity. In a different planning task, carried out by Laumann (2004), studying restorative effects of a walk in nature after a preceding heavy cognitive workload session with planning work, the pattern of cardiovascular results also coincided with the present data with respect to blood pressure, heart rate, baroreflex sensitivity and HRV.

The most important aspect of the studies mentioned above is the cognitive demand these place on the participants. For comparison, in a study on visual fatigue, Veldman et al. (1998) found partly comparable and partly different results. They studied cardiovascular changes during a 2.5 h lasting editing and error-correction task (visual work). During the first part of this task exactly the same pattern of results was found as in the present task. The pattern in the second part however, showed the opposite: blood pressure returned to original levels, while heart rate increased and baroreflex sensitivity decreased to starting levels.

Unfortunately, there are only very few studies in which blood pressure is measured continuously during driving or comparable tasks. The reasons are clear: both hands are needed for steering and controlling the car. For the current study we used a driving simulator with automatic transmission, keeping one hand available for finger blood pressure measurements. After a short training period of 10 min participants had no trouble with controlling the vehicle and were used to the blood pressure measurement. The data of the present car driving study completely resemble the results of two laboratory studies reported in Mulder et al. (1993), including the initial strong rise of blood pressure followed by a gradual decrease and a (continuing) relative small decrease of baroreflex sensitivity. In both studies a fast-paced memory search task was included (without counting) lasting for 45 min. Several other studies, where no blood pressure was measured, show the heart rate and HRV pattern of the present study (an initial increase of HR and a decrease of HRV, followed by a gradual decrease of HR and an increase of HRV). In a 1.5 h lasting car-driving study on the road, testing vigilance, De Waard and Brookhuis (1991) found a gradual decreasing heart rate (of about 8–10%) and a corresponding increase in HRV. These results suggest that the experienced workload and related invested effort in the current driving simulator might have been very low as well. The similarity of effects in the current study seems to be confirmed by the effects on reported mental effort.

In an overview, Backs and Boucsein (2000) list studies in which heart rate decreases as a function of time-on-task, sometimes in combination with an increasing HRV. Such studies include, for instance, monotony in train drivers (Myrtek et al., 1994), prolonged city bus driving (Milosovic, 1997) and long-haul bus driving (Raggatt and Morrissey, 1997). It must be noted that in most of these studies this pattern is considered to be connected to vigilance, diminished arousal or fatigue. Moreover, it has to be mentioned that this HR(V) pattern does not distinguish between the cardiovascular patterns of the present driving task and the ambulance planning task.

In conclusion, the above shows that results similar to those found in the dispatcher task are found in studies that are very comparable in nature to this planning task, while similar results to the driving task are found in other visual demanding tasks and other driving studies. As may be expected, this suggests that the nature of the tasks determines the cardiovascular response patterns to a great extent.

### Baroreflex model simulations

The next question to be answered is in which way the results of the present two studies can be characterized in terms of autonomic activation and short-term blood pressure (baroreflex) control. We performed a simplified simulation study using a baroreflex model for this purpose. This simplified procedure worked very well for the present data set, although it has of course restrictions with respect to the accuracy of the estimates. Complete simulations would have obtained better results but the present approach is good enough to describe patterns of autonomic activation in simulated task environments.

In terms of autonomic activation, the main differences between the two task situations can be summarized as follows: in the driving task the initial sympathetic activation is higher than in the ambulance planning task, while this activation as a function of time on task reduces during driving and increases during ambulance planning. This pattern completely corresponds with the blood pressure pattern. The main difference, however, is seen in vagal activation, which is decreased during driving and strongly increased during ambulance planning. The differences are clearly reflected in baroreflex sensitivity, being at a high level during the planning task and at a low level during driving.

## Conclusion

Looking at the overall results, one might conclude that there are distinctly different response patterns. For the ambulance planning task this pattern is very consistent over a series of studies, while this still has to be confirmed in future experiments for the driving task. The basic differences between the tasks are at the level of working memory and planning in the dispatcher task and active control (steering) in the driving environment. The difference in response patterns may perhaps be explained by looking at the level of control in the two tasks. Rasmussen (1987) differentiated between different levels of control: skill-based, rule-based and knowledge based. According to him, skill-based task performance requires fast and almost effortless processing, in contrast with knowledge-based performance, which requires slow, serial and effortful processing. Within the context of driving, Michon (1985) made a distinction between decision making at the tactical and operational level, which would correspond to knowledge-based and skill-based, respectively. An important difference between the two current studies is that the demands between intersections in the driving simulator task are mainly on an operational or skill-based level whereas those in the dispatcher task are most of the time on the knowledge-based or tactical level. There are, however, no conclusive arguments why this aspect would completely explain these large differences in response patterns. One possible additional explanation is that the dispatcher task gives such a high, continuing workload, resulting in an ongoing increase in blood pressure, that baroreflex is activated as strongly as possible in an effort to reduce blood pressure (Julius, 1988).

The question is what this means for applications in adaptive automation. With respect to the time duration of the working period, one could imagine that health consequences may build up in case an operator continuously goes on with working hard, having corresponding increases in blood pressure in combination with a maximal functioning baroreflex, for many hours as may occur in the ambulance planning task. For operators, doing this kind of work every day, it might be helpful or even necessary to reduce workload, for instance by having a “digital companion” that helps to reduce working memory activity. Although, in the short term this may not really help to reduce blood pressure, in the long run it might become important for keeping blood pressure within healthy boundaries.

The present (state) data are not really sensitive to changing short-term task demands; it is advised to use more short-term variables, such as HRV, RSA and respiratory rate for overload estimation. In this way overload might be detected at a short time-scale and task load can be adapted on the basis on such variables in combination with task performance measures.

What is clear from these two studies is that the effects of time-on-task, or more specifically on the blood pressure regulation mechanisms that cause these effects, have a large influence on the studied measures. When the measures studied in this paper, have to be applied for workload assessment, it has to be recognized that a substantial part of the effects observed may be related to short-term blood pressure control and not always directly related to workload manipulations. To find useful indices of mental workload (and invested effort) for applications in adaptive automation, it is necessary to take into account that the effects of time-on-task may actually overshadow the effects of workload manipulations. Success in adaptive automation based on psychophysiological measures may depend on the development of measures that are more sensitive to workload manipulations.

Furthermore, although different tasks elicit different effects, consistency within tasks is very high. Therefore, it can be concluded that these measures give insight in the cardiovascular effects of complex task performance but must be studied within the environment they are to be used in. Measuring and analyzing blood pressure next to heart rate is very helpful if not necessary, in understanding these cardiovascular patterns.

### Conflict of interest statement

The authors declare that the research was conducted in the absence of any commercial or financial relationships that could be construed as a potential conflict of interest.
